# Correlations of Valiltramiprosate Effects on Hippocampal Volume and Cortical Thickness with Clinical Outcomes in the Pre‐Specified MCI Group: Subgroup Analysis from the 78‐Week APOLLOE4 Phase 3 Trial in APOE4/4 Homozygotes

**DOI:** 10.1002/alz70861_108827

**Published:** 2025-12-23

**Authors:** Susan Abushakra, P. Murali Doraiswamy, Earvin Liang, Duygu Tosun, Marwan N. Sabbagh, Anton P. Porsteinsson, David Watson, Aidan Power, Sharon Cohen, Emer MacSweeney, John A. Hey, Susan Flint, Patrick Kesslak, Luc Bracoud, Chris Conklin, Adem Albayrak, Margaret Bray, Martin Tolar

**Affiliations:** ^1^ Alzheon, Inc., Framingham, MA USA; ^2^ Neurocognitive Disorders Program, Department of Psychiatry, Duke University School of Medicine, Durham, NC USA; ^3^ Alzheon Inc., Framingham, MA USA; ^4^ Center for Imaging of Neurodegenerative Diseases, San Francisco Veterans Affairs Medical Center, San Francisco, CA USA; ^5^ Barrow Neurological Institute, Phoenix, AZ USA; ^6^ University of Rochester School of Medicine and Dentistry, Rochester, NY USA; ^7^ Alzheimer's Research and Treatment Center, Wellington, FL USA; ^8^ Toronto Memory Program, toronto, ON Canada; ^9^ Re‐Cognition Health, London, Greater London UK; ^10^ Alzheon, Framingham, MA USA; ^11^ Clario, Inc., Lyon France; ^12^ Clario, Inc., Philadelphia, PA USA

## Abstract

**Background:**

Valiltramiprosate (ALZ‐801), an oral inhibitor of amyloid oligomer formation, was evaluated in a Phase 3 trial in APOE4/4 homozygotes with Early AD (Abushakra, 2024); the topline results being presented at this meeting (Power, 2025). While the primary clinical endpoint was not significant, hippocampal volume (HV, main imaging outcome) showed significant slowing of atrophy.

**Method:**

This 78‐week placebo‐controlled study randomized 325 homozygotes (placebo, 265 mg BID), stratified by MCI/Mild AD. ADAS‐Cog13 and CDR‐SB were the primary and key secondary outcomes respectively, and DAD (disability assessment for dementia) was a secondary outcome. HV was the main imaging outcome, cortical thickness and whole brain volume (CT, WBV) were secondary. Analyses by disease stage were pre‐specified using baseline severity as covariate in the MMRM model; drug‐effect correlations utilized Spearman’s correlations.

**Result:**

The pre‐specified MCI subgroup (MMSE 27‐30) included 125 subjects (58 placebo, 67 active); with balanced baseline characteristics except for cholinesterase‐inhibitors (placebo 31%, active 19%). Imaging population included 54 placebo, 62 active subjects. The MCI subgroup vs placebo showed positive effects on ADAS‐cog/DAD (nominal *p* <0.05), trend on CDR‐SB (*p* =0.053) with significant slowing of HV, CT and WBV atrophy vs placebo (26%, *p* =0.004; 35% *p* < 0.0001; 22%, *p* =0.027). In the active arm, change in HV correlated significantly with change in ADAS‐Cog13 (r=‐0.40, *p* <0.005), CDR‐SB (r=‐0.45, *p* <0.005), and DAD (r=0.33, *p* =0.018). For CT, the correlations were also significant: ADAS‐Cog13 (r=‐0.34, *p* =0.015), CDR‐SB (r=‐0.49, *p* < 0.005), and DAD (r=0.40, *p* <0.005). WBV treatment effect also correlated with the clinical outcomes (*p* ≤ 0.01).

**Conclusion:**

These strong correlations between structural preservation and clinical benefits of valiltramiprosate at the MCI stage support its efficacy and are consistent with its proposed mechanism of inhibiting amyloid oligomer formation, thereby potentially protecting neurons from synaptic dysfunction and neurodegeneration. Given the accelerated neurodegeneration in APOE4/4 patients, these findings suggest that intervention at the early stages of AD when neuronal reserve is higher, may be critical for valiltramiprosate to exert meaningful clinical effects alongside preservation of brain structure and volume.

